# Standardizing Clinical Workflow for Assessing Minimal Residual Disease by Flow Cytometry in Multiple Myeloma

**DOI:** 10.1016/j.clml.2022.10.008

**Published:** 2022-10-22

**Authors:** David M. Foureau, Barry A. Paul, Fei Guo, Edward H. Lipford, Kateryna Fesenkova, Elise Tjaden, Kendra Drummond, Manisha Bhutani, Shebli Atrash, Ami Ndiaye, Cindy Varga, Peter M. Voorhees, Saad Z. Usmani

**Affiliations:** 1 Immune Monitoring Core Laboratory, Levine Cancer Institute, Atrium Health, Charlotte, NC; 2 Department of Hematologic Oncology and Blood Disorders, Levine Cancer Institute, Atrium Health, Charlotte, NC; 3 Department of Hematologic Pathology, Atrium Health, Charlotte, NC; 4 Department of Cancer Biostatistics, Levine Cancer Institute, Atrium Health, Charlotte, NC; 5 Myeloma Service, Memorial Sloan Kettering Cancer Center, New York, NY

**Keywords:** Multiple myeloma MRD, Bone marrow collection, Multicolor cytometry assay performance, Reporting

## Abstract

To optimize specimen adequacy, our institution standardized technical 1st pull bone marrow aspirates (BMA) for minimal residual disease (MRD) testing in multiple myeloma. We are reporting assay performance characteristics from 556 MRD tests performed by flow cytometry. Ten million assay input was reached for 97.5% of tests, 76% were not hemodiluted, allowing us to routinely achieve 1–2 × 10^−6^ analytic sensitivity.

**Introduction:**

Minimal residual disease (MRD) status is an established prognostic biomarker for patients with multiple myeloma. Commonly used MRD testing techniques such as next generation sequencing or next generation flow cytometry can detect as little as one or two multiple myeloma plasma cells in 10^6^ normal bone marrow cells. Early pull of bone marrow aspirates (BMA), necessary to achieve such level of sensitivity, can be difficult to secure in routine clinical practice due to the competing need for early pull samples for clinical response assessment, therefore introducing the risk of analytical interference during MRD testing.

**Methods:**

To overcome this challenge, we standardized our workflow for collecting specimens by using a technical first pull after needle repositioning for MRD testing. To capture a comprehensive picture of MRD assay performance and specimen adequacy, we tested for MRD on 556 technical first pull bone marrow aspirates by next generation flow cytometry. Among the specimens, several key multiple myeloma treatment milestones were represented: end of induction therapy, two to three months post-autologous stem cell transplant, early and late stages of maintenance therapy.

**Results:**

By using the technical first pull bone marrow aspirate, we achieved an analytical assay input of 10 million nucleated cells for 97.5% of specimens. Our analytical sensitivity reached 10^−6;^ (i.e., 10 multiple myeloma plasma cells in 10 × 10^6^ bone marrow cells). Twenty-four percent of specimens were significantly hemodiluted. Low assay input or hemodilution quantifiably lowered the assay sensitivity.

**Conclusion:**

Specimen adequacy is, therefore, an important metric to incorporate into MRD status reporting.

## Introduction

Minimal residual disease (MRD) testing is accepted as an important test for when assessing disease response and prognosis in patients with multiple myeloma.^1 , 2^ MRD has also become a commonly reported endpoint for new drug development.^3–5^ Clinical trials are now exploring escalating and de-escalating treatment for multiple myeloma based on MRD status.^6 , 7^

The International Myeloma Working Group currently defines MRD as the persistence or re-emergence of very low levels of cancer cells – approximately 1 tumor cell in ≥ 10^5^ normal bone marrow cells (ie 1 × 10^−5^ sensitivity) – in patients who have achieved complete remission.^8^ Among the most commonly used techniques for detecting MRD for multiple myeloma are next generation sequencing (NGS) and next generation flow cytometry (NGF), which can reach even higher sensitivity levels in standard practice to detect as little as 1 to 2 tumor cell(s) in 1 million normal bone marrow cells (1–2 × 10^−6^ sensitivity).^9^ Both techniques have been extensively validated by multiple institutions and show strong concordance in measurements.^10–12^ The lab testing workflow for MRD NGF testing is fully standardized for assay input, antibody panel, and data analytics.^13–16^

As the assays used for detecting MRD are becoming increasingly more sensitive, the quality of bone marrow aspirate used can profoundly impact results. Hemodilution and low assay input are common issues that can negatively affect MRD testing accuracy.^17^ Using an early pull bone marrow aspirate for MRD testing can circumvent these issues. This often conflicts with standard clinical workflow for clinical response assessment, also requiring early pull bone marrow aspirates. For instance, establishing a stringent complete response requires < 5% plasma cells in the bone marrow and the absence of clonal cells as detected by either immunohistochemistry or immunofluorescence, according to International Myeloma Working Group criteria.^8^

To overcome this challenge, we standardized our bone marrow aspirate specimen collection workflow by systematically assigning MRD testing to a technical first pull after needle repositioning. This strategy allows us to collect early pull bone marrow aspirates for both clinical response assessment and MRD testing. Here, we present assay performance metrics, key assay input considerations for MRD NGF testing from technical first pull bone marrow aspirates, and data reporting strategy that incorporates specimen quality assessment.

## Methods

### Specimen procurement.

Patients with multiple myeloma were enrolled in a prospective observational study under an IRB-approved specimen collection protocol for plasma cell disorders between 2016 and 2020. Bone marrow aspirates were collected with or with radiologic guidance using heparinized syringes at key treatment milestones: the end of induction therapy, 2 to 3 months post-autologous stem cell transplant, early (1–2-year post-ASCT) and/or late (3–8 years post-ASCT) maintenance therapy. During each bone marrow aspirate procedure, the first needle position (pull #1–3) was used to collect bone marrow aspirate for standard plasma cell disorder lab tests (Table 1). We collected bone marrow aspirate for MRD testing after needle repositioning as a technical first pull. Two 2 to 3 mL of bone marrow aspirate, which was subject to a bevel turn/twist of 45 ° in between, was placed into vacutainer tubes coated with K_2_EDTA. The bone marrow pull number was documented at the time of specimen collection on a specimen requisition form and each tube collected. All MRD assays were performed on the same day as specimen collection.

### MRD testing and evaluation.

MRD testing was performed by NGF using a standardized 2 tubes/ten marker multicolor flow cytometry technique.^15^ Briefly, the MRD NGF panel utilizes 8 surface markers (CD38, CD138, CD19, CD56, CD45, CD81, CD117, CD27) and anti-cytoplasmic immunoglobulin light chain (kappa and lambda) antibodies to distinguish normal plasma cells (N-PCs) abnormal/clonal multiple myeloma plasma cells (MM-PCs). Following bulk red blood cell lysis of bone marrow specimen collected for MRD testing, 2 aliquots of 1 × 10^7^ cells each were stained with surface antibody markers (all 8 surface markers for 1 aliquot and 6 – excluding CD81 and CD117 - for the second aliquot) for 30 minutes. Following cell fixation and permeation of both aliquots, intracellular staining for cytoplasmic immunoglobulin light chain kappa/lambda was performed on cells of the second aliquot for 15 minutes. At least 5 × 10^6^ events from each aliquot were acquired for analysis on a BD lsrFortessa flow cytometer (BD Biosciences, CA). Infinicyt (Cytognos, Spain) was used to merge the 2 fcs files generated for each assay for analysis.

Absolute MM-PC distribution and MRD status at both 10^−5^ and 10^−6^ virtual sensitivity were prospectively recorded. Quality metrics bone marrow aspirate specimens were also captured prospectively. Post-red blood cell lysis cell count and viability was measured using the Muse Count Viability kit a Muse Guava cell analyzer as per manufacturer’s instruction (Luminex, TX). Acquired specimen hemodilution was assessed using mast cell distribution (CD19^−^, CD38^low^, CD117^high^).

### Statistics.

Descriptive statistics were obtained using Prism (GraphPad, CA). The lower limit of normal of mast cell distribution was calculated at each clinical time point: Lower limit of normal = mean mast cell frequency - 1 standard deviation. Analysis of variance (ANOVA) was used to test for differences in nucleated cell counts, mast cells, normal PCs and MM-PCs in bone marrow aspirates across clinical time points. Contingency analyses (Chi-squared tests) were used to evaluate MRD status differences between clinical time points.

The performance of MRD NGF assays on technical first pull bone marrow aspirates was established by calculating background, analytical, and functional sensitivity.^18^ For each targeted virtual MRD sensitivity value, a virtual mean of blank (_v_x= Blank) and corresponding standard deviation (_v_*σ* - Blank) was calculated using absolute quantification values of MM-PC detected below the set virtual sensitivity threshold (ie MRD negative samples). Assay background (limit of blank, LOB) was defined as the highest apparent analyte concentration expected to be found when MRD negative samples are tested (LOB = _v_x= Blank + 1.6_v_*σ* - Blank). Assay analytical sensitivity (limit of detection, LOD) was defined as the ability to detect the MRD at a level that can reliably be distinguished from the LOB, where 95% of low levels samples will be detected above the LOB (LOD = _v_x= Blank + 3_v_*σ* - Blank). Assay functional sensitivity (lower limit of quantification, LLOQ) was defined as the lowest concentration that can be reliably detected with acceptable accuracy and precision (LLOQ = _v_ x= Blank + 10_v_*σ* - Blank). Root-mean-square error was used to measure the differences between calculated and targeted LOD values.

### Data sharing statement:

original data can be made available in response to a reasonable, written request to the corresponding authors.

## Results

### Cohort description.

We performed 556 MRD assays on bone marrow aspirates collected from 307 patients (Table 2). Only 10.6% of samples (59 of 556) were from CT-guided biopsies; the remaining were collected from the iliac crest at the bedside. Most MRD assessments (76.8%) took place in the first year following diagnosis and treatment. Approximately half of patients (51%; 156 of 307) were tested at 2 or more clinical time points. At the time of MRD assessment, 88% of the patients had achieved a very good partial response or better (stringent complete response/complete response: 50%, very good partial response: 38%).

Among patients tested for MRD before maintenance therapy (ie either after induction therapy or 60 to 90 days after autologous stem cell transplant [ASCT]), the main induction regimens received were triplet proteasome inhibitor (PI)-Immunomodulatory drug (IMiD)-dexamethasone (Dex) (72%) and PI-cyclophosphamide (Cy)-Dex (10%). Twenty-nine patients received daratumumab-containing induction regimens. Among patients tested during maintenance therapy (between one and 8 years post-ASCT), single agent IMiD was the most common maintenance regimen (60%), followed by PI only (16%).and PI-IMiD (14%).

### Residual disease dynamic throughout standard of care for multiple myeloma.

**Figure 1:**
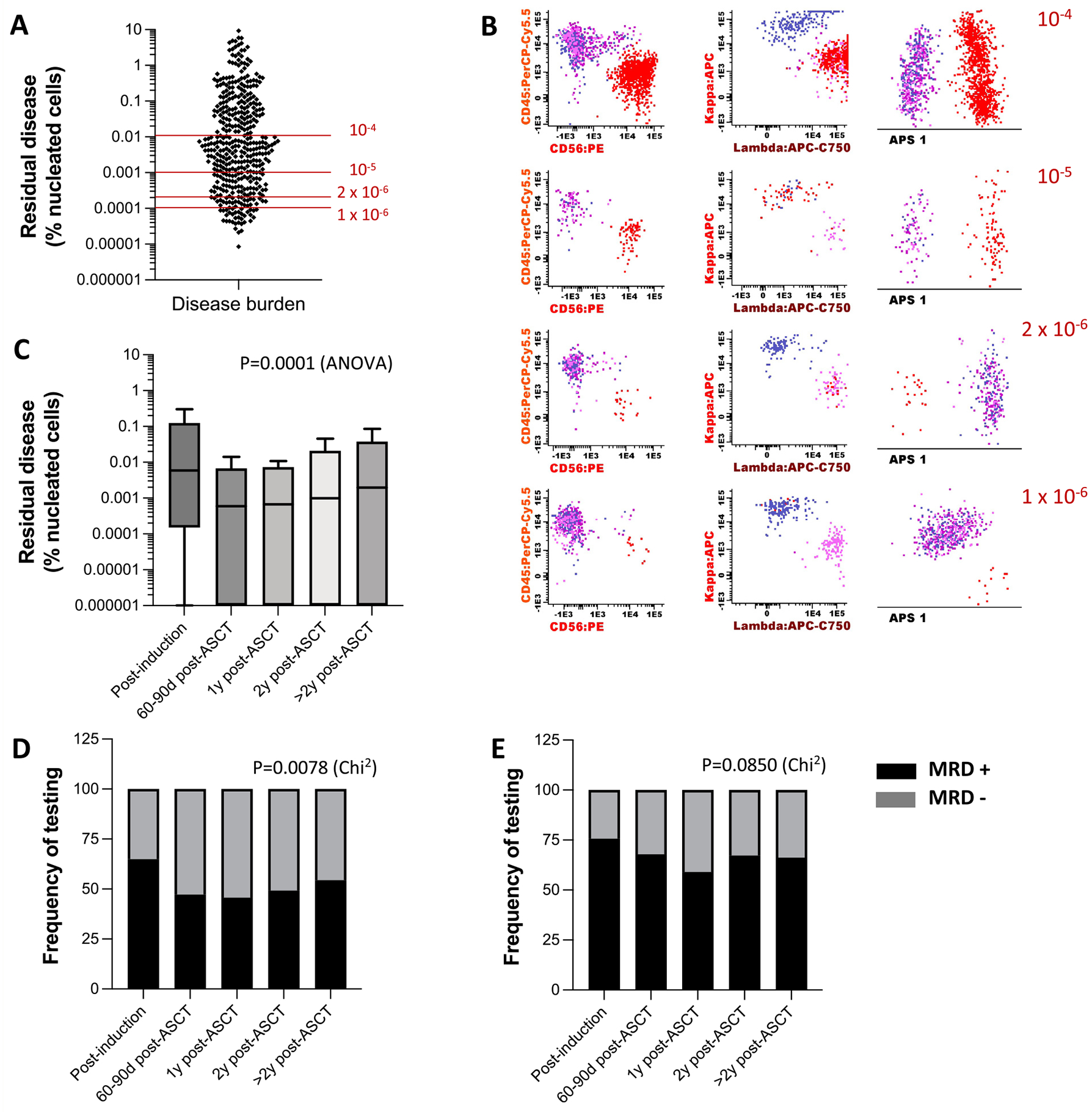
Disease Burden and MRD status changes throughout standard of care. Disease burden was measured for 556 bone marrow aspirate samples collected from 307 patients. A. Disease burden distribution among specimens with detectable abnormal/clonal plasma cells (ie MM-PCs). B. Representative data output for disease burden as represented by 1 × 10^−4^ to 1 × 10^−6^ of total nucleated cells (gated from CD19- CD56_high_ plasma cell subset). C. Tukey representation of residual disease burden across clinical time points. Boxes represent median and interquartile distances. Whiskers represent non-outlier farthest measurements. D-E. MRD status across clinical time points at (D) 1 × 10^−5^ sensitivity and (E) 1 × 10^−6^ sensitivity

We tested the technical first pull bone marrow aspirate for MRD by NGF and found that disease burden varied among patients, with a median MM-PC distribution of 0.0013% (range, 0%–9.3%) of bone marrow nucleated cells. Overall, 30% of patient samples were MRD positive at 1 × 10^−4^ virtual sensitivity, 52.5% at 1 × 10^−5^, 64.4% at 2 × 10^−6^, and 69% at 1 × 10^−6^ (Figure 1A). Thirty-four additional specimens presented with an ultra-low frequency (< 1 × 10^−6^) of MM-PCs. Phenotypic/clonality assessment and principal component analysis (APS1 diagram) clearly distinguished normal PCs from MM-PCs regardless of the MRD virtual sensitivity threshold (Figure 1B).

Our analyses of MM-PC distribution revealed that the specimen sampling timeline of individual patients constituted an important source of disease burden variability and reflected disease course. Post-induction therapy, patients presented with disease burden on average 10-fold higher than 60 to 90 days post-ASCT (0.0059 vs. 0.0005%, *P* < .0001) (Figure 1C). While median disease burden detected 1-year post-ASCT remained low (0.0007%), it gradually increased in subsequent years (*P* = .0001). When we assessed MRD status at 10^−5^ virtual sensitivity, a similar trend was observed; only 35% of patients were MRD negative after induction therapy, 55% by 1-year post-ASCT (Figure 1D). The MRD negativity rate gradually decreased by an average of 4% yearly thereafter (*P* = .0078). However, when we used a 10^−6^ virtual sensitivity, our results revealed a noticeably different trend in which 24% of patients reached MRD negativity post-induction and one third of these patients remained MRD negative thereafter (*P* = .0850) (Figure 1E).

### Characteristics of technical first pull bone marrow aspirate for MRD NGF.

**Figure 2:**
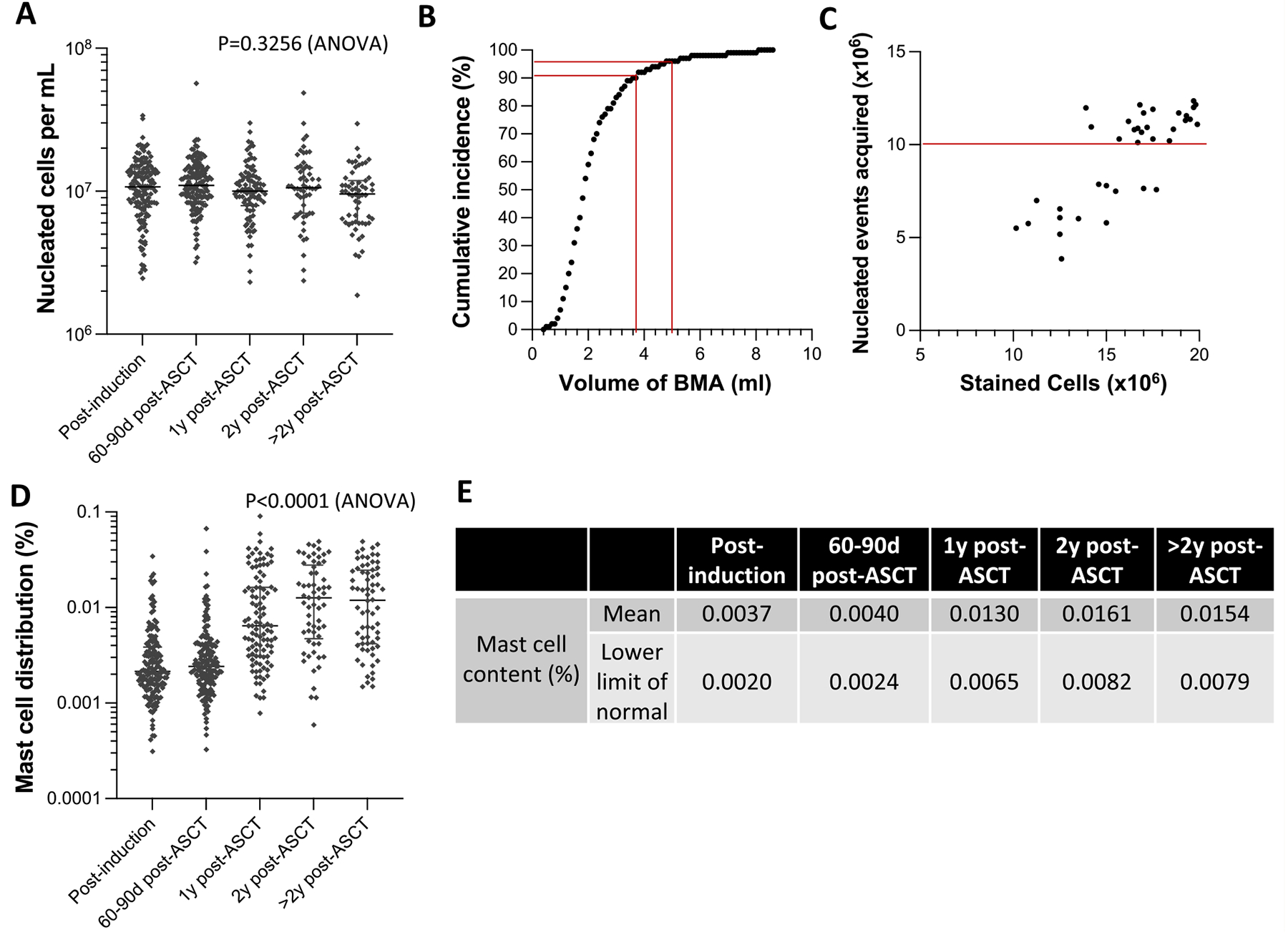
Characteristics of first technical pull samples of bone marrow aspirate. Quality control metrics were prospectively recorded for all specimens. A. Specimen cellularity distribution across clinical time point, bars represent mean ± SEM. B. Cumulative incidence of tests reaching 20 × 10^6^ cells input as volume of bone marrow aspirate collected increase. Red bars indicate volume of bone marrow aspirate required to achieve 20 × 10^6^ for 90% and 95% of samples, respectively. C. Number of events acquired for MRD tests with an assay input < 20 × 10^6^ cells, *i.e*. 37 of 556 (6.6%) of MRD assays evaluated on our study. The red bars mark 10 × 10^6^ events acquired; the minimum analytical input required to make a MRD determination at 1–2 × 10^−6^ sensitivity by NGF. D-E. Percentage of mast cells in bone marrow cells across clinical time points with the corresponding lower limit of normal values

To achieve the highest level of assay sensitivity, the recommended input for MRD testing by NGF is 20 × 10^6^ cells; 10 × 10^6^ cells stained for surface markers only (tube A) and 10 × 10^6^ cells stained for both intracellular markers and cytoplasmic immunoglobulin light chains (tube B). In our study, the average cellularity of technical first pull bone marrow aspirate for all 556 samples was 11.5 ± 7.7 × 10^6^ cells per mL and on average, with 90.9 ± 9% of cells remaining viable post-red blood cell lysis. We observed no significant differences in the bone marrow aspirate cellularity across clinical time points (*P* = .3256) (Figure 2A). In 90% of cases, 3.6 mL of technical first pull bone marrow aspirate was required to obtain the 20 × 10^6^ input cells; in 95% of cases, 4.7 mL (Figure 2B). Additional volume of bone marrow aspirate beyond 4.7 mL only incrementally increased the cell count.

The recommended analytic input for MRD NGF, to reach the highest level of assay sensitivity, is at least 10 × 10^6^ nucleated events (≥5 × 10^6^ from tube A and ≥5 × 10^6^ from tube B).^14 , 15^ For only 6.6% of samples (37 of 556), we were unable to reach the desired assay input of 20 × 10^6^ cells (Figure 2C). For 26 of these samples, we were able to stain at least 15 × 10^6^ cells and successfully collected ≥ 10 × 10^6^ nucleated events more than 80% of the time (21 o 26 samples). The remaining 11 samples had less than 15 × 10^6^ stained cells and we successfully collected ≥ 10 × 10^6^ nucleated events for only 2 (18%) of these samples.

Beyond assay input, specimen hemodilution is another important limiting factor that can impact MRD assay sensitivity. Hemodilution can be evaluated by MRD NGF by measuring the distribution of bone marrow resident leukocytes that are absent in peripheral blood. For this purpose, we prospectively captured mast cell content from the technical first pull bone marrow aspirate. Among the 556 samples that we analyzed, mean mast cell content was 0.0083 ± 0.0113% bone marrow nucleated cells. Mast cell content varied significantly between different clinical time points (Figure 2D). Overall, mast cell content was lower during early treatment (0.0038 ± 0.0056%; post-induction and 60 to 90 days post-ASCT) than at later clinical time points (0.0145 ± 0.0139; ≥ 1-year post-ASCT) (*P* < .0001). The lower limit of normal (LLN) mast cell content in technical first pull bone marrow aspirate also varied widely: average 0.0048% (range, 0.0020% [post-induction] - 0.0082% [2 years post-ASCT]; Figure 2E). Within the context of our bone marrow collection standard operating procedure, we did not observe any correlation between aspirate volume and mast cell content < LLN compared with ≥LLN (4.9 ± 1.3 vs. 5.0 ± 10.3 mL; *P* = .2305).

Twenty-nine patients enrolled in our study received a daratumumab-containing regimen within 12 months of MRD assessment (Table 2). All achieved the required 10 million events analytical input. The average mast cell content of bone marrow specimen collected from daratumumab treated patient was 0.007 ± 0.009%, 2 patients presented with < 0.002%. Overall, 15 (51.7%) patients tested MRD positive at 1 × 10^−5^ and 17 (58.6%) at 1 × 10^−6^ sensitivity. Both normal and abnormal plasma cell populations displayed a key phenotypic difference between daratumumab treated and naïve patients: CD38 staining intensity was significantly lower (yet still elevated) following daratumumab exposure (mean fluorescence intensity 15,913 ± 18,379 vs. 74,294 ± 17,622; *P* < .0001). Both plasma cell populations retained high CD138 expression.

### Assay performance characteristics for MRD NGF testing of technical first pull bone marrow aspirate.

The Euroflow consortium established the analytical sensitivity (LOD) of NGF assay to as 2 × 10^−6^ (20 MM-PC cells in 10 × 10^6^ bone marrow cells) and functional sensitivity (LLOQ) as 5 × 10^−6^ (50 MM-PCs in 10 × 10^6^ bone marrow cells).^1^ To confirm that using a technical first pull of bone marrow aspirate for MRD NGF testing did not negatively impact assay performance, we re-evaluated assay background, analytical and functional sensitivity.

**Figure 3:**
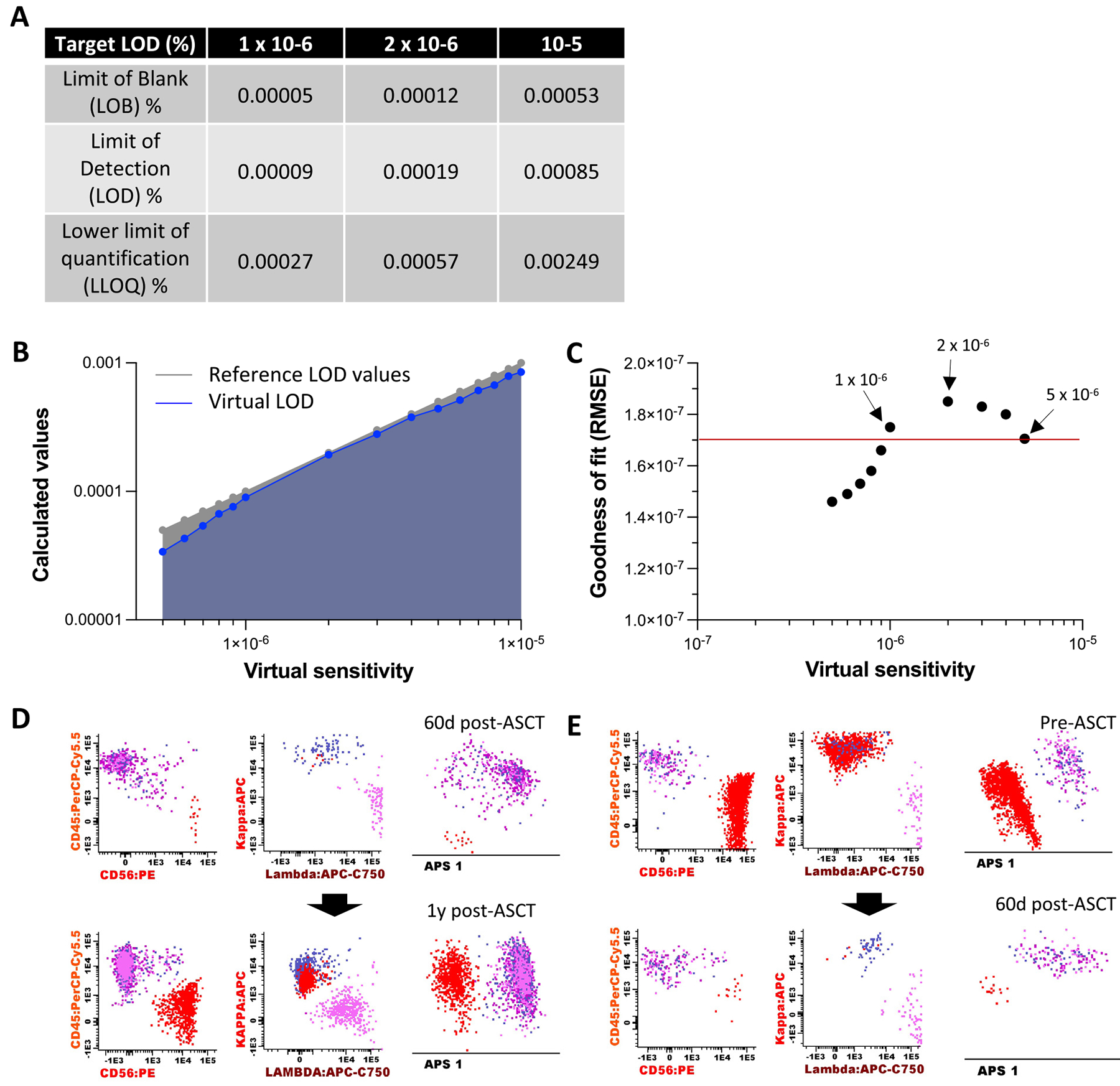
Performance of MRD assay by NGF. Background, analytical, and functional sensitivity were calculated at various level of targeted assay sensitivity ranging from 10^−5^ to 0.5 × 10^−6^. A. Limit of blank (LOB)/limit of detection (LOD)/ and lower limit of quantification (LLOQ) values at 1 × 10^−6^, 2 × 10^−6^ and 1 × 10^−5^ targeted sensitivity. B. Limit of detection (LOD) across targeted sensitivities ranging from 1 × 10^−5^ to 0.5 × 10^−6^, in 0.1 log increments are represented on the right. In blue are calculated virtual LOD values plotted against targeted LOD; in grey are reference values where targeted LOD values would match virtual LOD. C. Goodness of fit (using root-mean-square error, RMSE) between plotted virtual LOD (vLOD) values and reference LOD value at different level targeted MRD virtual sensitivity threshold. The red line indicates goodness of fit at 5 × 10^−6^ sensitivity. D. Representative case of a patient who transitioned from MRD^low^ (10–19 MM-PCs in 10 × 10^−6^ bone marrow cells) to MRD^high^ (> 100 MM-PC in 10 × 10^6^ bone marrow cells) during the first year of maintenance therapy. E Representative case of a patient who showed an improved response from MRD^high^ pre-ASCT to MRD^low^ post-ASCT

As we had successfully acquired 10 × 10^6^ events for > 98% of our samples (Figure 2C), we established a targeted sensitivity for the assay at 2 × 10^−6^ . By compiling MM-PC distribution in MRD negative specimens at this sensitivity threshold, we calculated the following assay performance: LOD = 0.00019 and LLOQ = 0.00057 (Figure 3A). In 43 specimens, we detected MM-PCs below this threshold (7.8%), which suggests that a higher sensitivity could be achieved from technical first pull bone marrow aspirate. Therefore, we further explored assay performance by calculating virtual LOD values at targeted assay sensitivities ranging from 1 × 10^−5^ to 0.5- × 10^−6^ (our limit at which a cluster of abnormal/clonal MM-PCs can be reproducibly resolved) in 0.1 log_10_ increments (Figure 3B). We measured virtual LOD values goodness of fit against reference LOD values (ie targeted virtual sensitivity thresholds) using root-mean-square error (Figure 3C). Between virtual sensitivity thresholds of 5 × 10^−6^ and 1 × 10^−6^, root-mean-square error values of virtual LOD against targeted LOD remained ≥ 1.705 × 10^−7^. At 0.9 × 10^−6^ virtual sensitivity threshold root-mean-square error values dropped to 1.660 × 10^−7^ and further decreased thereafter. Taken together, our data indicate that a 1 × 10^−6^ sensitivity can be achieved by MRD NGF on the technical first pull bone marrow aspirate; we calculated assay performance as LOD = 0.00009 and LLOQ = 0.00027.

To confirm the ability of MRD NGF performed on a technical first pull bone marrow aspirate to reliably detect as few as 10 MM-PCs in 10 × 10 ^6^ bone marrow nucleated cells, we identified samples from patients who had serial MRD assessments and identified those with MRD that transitioned between MRD^low^ (10–19 MM-PCs) and MRD^high^ (> 100 MM-PCs) status within a 12-month period. Five patients went from MRD^low^ to MRD^high^ during the first year of maintenance therapy (Figure 3D). A further 6 patients went from MRD^high^ to MRD^low^ between the end of induction therapy and 60 to 90 days post-ASCT (Figure 3E). In all cases (11 of 11), MM-PCs displayed phenotypic and clonal concordance between MRD^low^ and MRD^high^ measurements suggesting the false MRD^low^ positivity rate was 0% for those 11 patients. Another 5 patients transitioned from MRD^high^ to MRD^ultra-low^ (5–9 MM-PCs per 10 × 10^6^ bone marrow cells) between the end of induction and 60 to 90 days post-ASCT (data not shown). While 3 patients had the same MM-PC population between MRD assessments, we found phenotypic and clonal discordance for 2 patients, suggesting a false MRD^ultra-low^ positivity rate of at least 40% for those 5 patients. Therefore, these data confirm that, residual disease was indeed reliably detectable at ≥ 1 × 10^−6^ sensitivity by MRD NGF on the technical first pull bone marrow aspirate. Below this threshold, the risk of false positivity rises significantly.

### Reporting MRD status on suboptimal technical first pull bone marrow aspirate.

While rare in our study, low assay input interfered with our ability to detect MRD at the highest level of sensitivity of the assay for 14 of 556 samples. Our data indicate that in most instances, as few as 15 × 10^6^ stained cells were sufficient for acquiring 10 × 10^6^ events (Figure 2C); therefore, the adequacy of the assay input for detecting MRD should be based upon the number of acquired events (analytical input) rather than the number of cells stained. For specimens that did not meet the recommended analytical input requirements, 9 (64.3%) were MRD positive. For the remaining 5 specimens, we calculated a revised LOD value (revised LOD = 10 / analytical assay input) and established MRD negativity at sensitivity ranging from 1.171 × 10–6 to 2.589 × 10^−6^.

**Figure 4:**
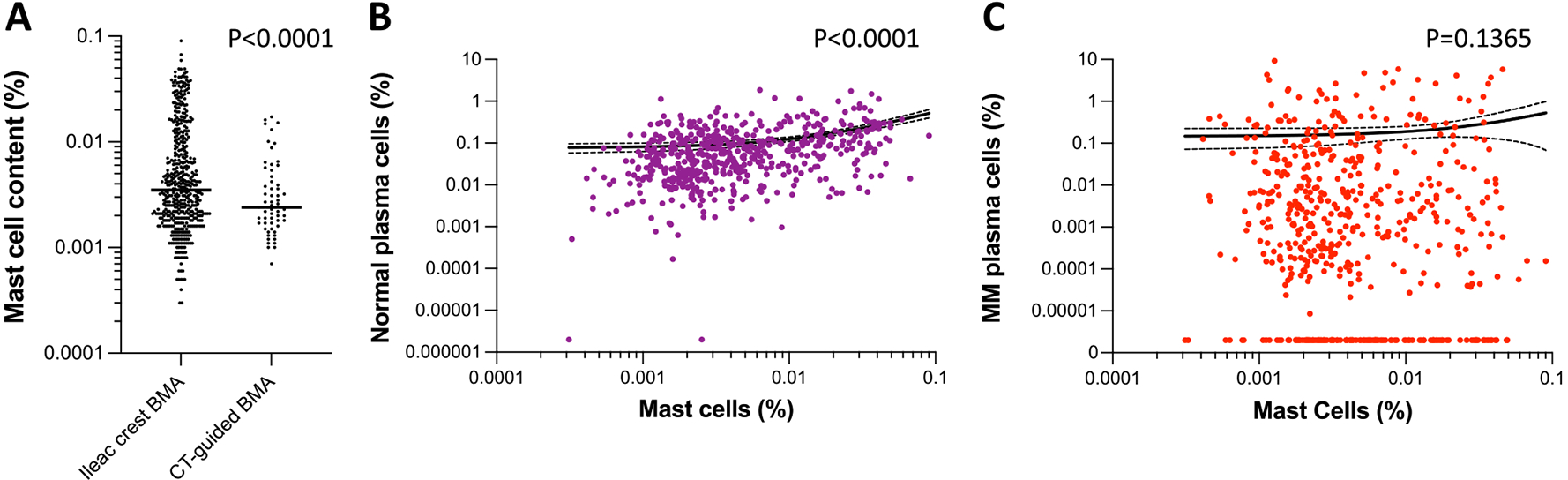
Impact of hemodilution on MRD detection. Mast cell content in bone marrow aspirate was plotted against disease burden and MRD status. A. Mast cell distribution in bone marrow aspirate collected by iliac crest bone marrow biopsy vs. CT-guided bone biopsy. B. Regression analyses of mast cell content relative to normal PC content and C. Regression analyses of mast cell content relative to MM-PC distribution

Hemodilution was a more common issue than low assay input. Using mean mast cell content and lower limit of normal to define specimen adequacy, we determined that 213 of 556 specimens were adequate (≥ 0.0048% mast cells); 209 were marginally adequate (≥ 0.002% mast cells); and 134 were suboptimum for MRD testing due to hemodilution. The procedure that we used to collect the technical first pull bone marrow aspirate did impact hemodilution levels. Samples collected via an iliac crest bone biopsy at the bedside had lower levels of hemodilution than those collected by CT-guided biopsy (0.008 ± 0.01 vs 0.004 ± 0.004% mast cells, *P* < .0001) (Figure 4A). We investigated whether hemodilution impacted normal PC distribution and confirmed a significant decrease that coincided with diminishing mast cell content (*P* < .0001) (Figure 4B). When we performed the same analysis with MM-PCs, we also observed a similar trend; however, the decrease was not considered statistically significant (*P* = .1365) (Figure 4C). Importantly, among the 134 specimens that were significantly hemodiluted, residual disease was detected in 96 of them. Taken together, these data suggest that while MM-PCs may remain detectable in some hemodiluted specimens, disease burden may be underestimated, and assay sensitivity lowered.

## Discussion

At our institution, we collect the bone marrow aspirate for MRD testing from the technical first pull as part of standard clinical procedures. To determine the performance of NGF assay for measuring MRD with the technical first pull bone marrow aspirate, we tested samples from patients that represented key treatment milestones: end of induction, 2 to 3 months post-ASCT, and during early and late maintenance therapy. To our knowledge, this study represents the largest dataset of MRD assays on technical first pull bone marrow aspirates.

We have shown that by using the technical first pull aspirates, MRD status can be determined at 10^−6^ sensitivity threshold (≥ 10 MM-PCs in 10 × 10^6^ bone marrow nucleated cells). While current guidelines set the target MRD sensitivity for multiple myeloma at ten times greater than this threshold and MRD status is commonly reported at 10^−5^ sensitivity, both next generation sequencing and NGF have reproducibly achieved 1–2 × 10^−6^ sensitivity.^8^ The Euroflow consortium established that MRD NGF assays should be performed at 2 × 10^−6^ analytical sensitivity (LOD) and 5 × 10^−6^ functional sensitivity (LLOQ).^1^ In our study, we calculated an assay performance of 1.9 × 10^−6^ LOD and 5.7 × 10^−6^ LLOQ matching Euroflow assay performance characteristics. In addition, we found that a higher level of sensitivity is achievable using first pull bone marrow aspirate as we were able to reliably detect as few as 10 MM-PCs in 10 million bone marrow cells (ie 1 × 10^−6^ sensitivity). However, routinely achieving such a level of sensitivity by MRD NGF remains technically challenging and requires strong adherence to standardized specimen collection procedures and NGF protocols. Results of a retrospective study published in 2020 showed that clinically meaningful differences existed between patients without MRD at either 10^−5^ or 10^−6^ sensitivity; those without MRD at 10^−6^ sensitivity had longer PFS.^19^ Findings from our study showed that during the first 2 years of maintenance therapy, the MRD negativity rate significantly decreased at 10^−5^ virtual sensitivity while MRD negativity rates remained stable at 10^−6^ sensitivity, suggesting that MRD negativity at 10^−6^ better represents the absence or residual disease. Achieving 1 × 10^−6^ sensitivity by MRD next generation sequencing (Clonoseq, Lymphotrack) typically requires an input of 30 μg DNA, the equivalent of 3 × 10^6^ nucleated cells.^20^ A larger analytic input is required for multicolor flow cytometry techniques such as MRD NGF to obtain the typical requirement of 10 × 10^6^ events.^15^ In more than 90% of our cases, 3.6 mL of technical first pull bone marrow aspirate met this requirement.

Hemodilution is a very important variable to consider when performing MRD testing for multiple myeloma as it can represent a very important potential sampling bias for both molecular and multicolor flow cytometry MRD techniques. Greater assay sensitivity places further importance on mitigating hemodilution issues.^3^ At 10^−5^ sensitivity, MRD next generation sequencing or NGF can detect > 30 MM-PCs in 3 × 10^6^ bone marrow cells and > 100 MM-PCs in 10 × 10^6^ bone marrow cells. Lowering the threshold for MRD positivity to 10^−6^ brings those numbers ten times lower, to > 3 MM-PC in 3 × 10^6^ bone marrow cells and > 10 MM-PC in 10 × 10^6^ bone marrow cells and suggests that hemodilution could greatly impact the ability to detect MRD at this sensitivity level. When multicolor flow cytometry techniques are used for assessing MRD in multiple myeloma, it is therefore important leverage this technology to test for hemodilution. Puig et al recently published comprehensive reference values for the B-cell precursors, mast cells and nucleated red blood cells, which are bone marrow resident cell subsets that are commonly used for evaluating bone marrow aspirate hemodilution.^21^ In their analyses of samples from 65 patients with multiple myeloma, Puig and colleagues found that median mast cell content detected in bone marrow cells was 0.004% (Q25 0.001) following an induction regimen of PI-IMiD-Dex (bortezomib, lenalidomide and dexamethasone); 0.0095% (Q25: 0) after a maintenance regimen of lenalidomide and low-dose dexamethasone.^21^ Granted our study was limited by the lack of a direct 1st pull comparator, the mast cell content that we are reporting is very similar to these reference values (0.0037 ± 0.0013% post-induction therapy; 0.0130 ± 0.0065% 1 year post-ASCT). Most patients enrolled in our study received PI-IMId-Dex induction therapy and IMiD maintenance therapy. While Puig et al. observed higher mast cell content in bone marrow aspirate collected following daratumumab, carfilzomib, lenalidomide and dexamethasone (daratumumbab with PI-IMId-Dex; 0.01%; Q25 0.004), we did not observe a similar trend for patients treated with daratumumab-containing regimens in our cohort.

Specimen hemodilution did not evenly impact normal PCs and MM-PCs. While normal PC levels declined proportionally to mast cell content, MM-PCs levels were relatively less affected by hemodilution. While our results do not offer a direct explanation for this observation, disease biology or a sampling bias could have partly contributed to this trend. Plasma cells preferentially reside in the bone marrow, and adhesion molecules such as CD44 glycoprotein or integrins are upregulated by MM-PCs.^22 , 23^ Therefore, stronger adhesion to the bone marrow extracellular matrix could be contributing to higher retention of MM-PCs following hemodilution. In addition, we observed that the bone marrow aspirates collected by CT-guided bone biopsy were more often hemodiluted than those collected by iliac crest biopsy. While other studies have shown that CT-guided bone biopsies tend to provide better quality bone marrow aspirate and, in the context of MRD testing, may yield specimens that are less impacted by hemodilution, our study suggested otherwise.^24^ The reason for this discordance is not clear at this time, but we suspect is at least partially explained by the variance in number of providers performing each procedure (with significantly more providers performing fewer procedures in radiology). Nevertheless, we observed that at a minimum, hemodilution affects assay sensitivity and should be taken into consideration when reporting MRD results. This is not to say those results should be dismissed outright though since a large portion of hemodiluted specimens contained detectable MM-PC. While disease burden is underestimated in such cases, the MRD positive status remains. An issue truly arises in the absence of detectable residual disease. Our data indicate that patients with disease burden ≥ 100 MM-PCs in 10 × 10^6^ analyzed bone marrow nucleated events (ie ≥ 1 × 10^−5^ disease burden) would retain detectable residual disease by MRD NGF, even for heavily hemodiluted specimens with up to 9-part blood for 1-part bone marrow. It is unclear though whether specimen with very low disease burden (≥ 10 but < 100 MM-PCs in 10 × 10^6^ bone marrow nucleated events) would be captured in the presence of hemodilution or a low assay input.

## Conclusion

An adequate technical first pull bone marrow aspirate – 10 × 10^6^ cells as assay input with no hemodilution – can routinely achieve 1–2 × 10^−6^ sensitivity by MRD NGF. A lower assay sensitivity can be achieved for limiting samples with either low assay analytical input (< 10 × 10^6^ cells) or presenting significant hemodilution (< 0.002% mast cells). Such limiting factors may not necessarily preclude MRD status reporting though, especially for MRD positive cases. However, it is key to emphasize the lower assay sensitivity when reporting MRD status for such cases.

## Figures and Tables

**Table 1:** Bone Marrow Aspirate Pull Order for Standard Labs in Multiple Myeloma

First Position Standard plasma cell disorder Labs - Pull order	Second Position MRD testing
First pull. **Morphology review** : 2–3 ml in heparinized syringe	**MRD testing.** To be performed if patient is in ≥ very good partial response: 2–3 ml in plain syringe into K2-EDTA tube × 1, rotate bevel 45°, aspirate an additional 2–3 ml in 2nd plain syringe into K2-EDTA tube × 1.
Second pull. **Multiple Myeloma FISH Panel** : 2 ml in plain syringe into EDTA tube × 1	
Third pull. **Standard plasma cell Flow** : 1–2 ml in plain syringe into EDTA tube × 1	
*Optional - ordering provider to specify if additional tests requested*	
***Cytogenetics:*** *2 ml in heparinized syringe*	
Preparation of heparinized syringe: Take up ~0.5 ml heparin and pull the plunger back to line the barrel.	
To reduce hemodilution, perform one aspiration per bevel orientation–turn/twist the bevel 45° for each subsequent pull from the same position (eg first position first pull: 1 ml for morphology, turn bevel 45°, first position second pull: 2 ml for FISH, etc.)	

**Table 2:** Specimen and Cohort Characteristics

Bone marrow aspirates (N = 556)	N (%)
Specimen collection	
Bedside iliac crest biopsy	497 (89.4)
CT-guided biopsy	59 (10.6)
Time of specimen collection	
Post-induction therapy	157 (28.2)
60–90 d post-ASCT	165 (29.7)
1 y post-ASCT	105 (18.9)
2 y post-ASCT	61 (11.0)
> 2 y post-ASCT	68 (12.2)
Clinical response at the time of MRD assessment	
CR/sCR	279 (50)
VGPR	205 (38)
< VGPR	33 (6)
NA	39 (7)
	
Patient’s treatment information (N=307)	%
Main induction therapy regimen	
PI-IMiD-Dex	72
PI-Cy-Dex	10
Daratumumab-containing regimen	5
Other/NA	13
Main maintenance therapy regimen	
Proteasome inhibitor only	16
IMiD	60
PI-IMiD	14
Other/NA	10

Abbreviations: ASCT = autologous stem cell transplant; CR = complete response; Cy = cyclophosphamide; Dex = dexamethasone; IMiD = immunomodulator; PI = proteasome inhibitor; sCR = stringent complete response; VGPR = very good partial response.

## Clinical Practice Points

- MRD status has been shown in several prospective and retrospective trials to be a reliable prognostic marker for myeloma patients treated with varying modalities. Increasingly, more studies are incorporating MRD status as a risk-adapted stratifier to test the power of MRD to function as a predictive biomarker.
- Currently, NGF MRD testing is only available in larger academic centers or at referral labs due to technical and logistical factors. As MRD testing becomes more ubiquitous and is incorporated in treatment decisions, the need to perform MRD testing at local institutions while still maintaining appropriate assay sensitivity and reproducibility will only become more important.
- Our paper details a protocol that allows for reliable MRD testing without compromising the fidelity of other bone marrow testing or subjecting patients to additional procedures or discomfort. We determined that collecting 3.6cc of bone marrow aspirate was sufficient to achieve a 10 million cell analytical input in 90% of cases.
- Further, our protocol incorporates mast cell quantitation as an internal control for hemodilution, allowing physicians to quickly assess the adequacy of the sample obtained and to interpret the data accordingly.
- If MRD does indeed have utility as a predicative biomarker, the ability to assess a patient’s MRD status quickly and reliably will only become more important for clinicians. We believe our assay shows that a technical first pull is a feasible, efficient, and reliable way of evaluating MRD status.
